# Single-Chain Mechanical Properties of Gelatin: A Single-Molecule Study

**DOI:** 10.3390/polym14050869

**Published:** 2022-02-23

**Authors:** Lu Qian, Kai Zhang, Xin Guo, Junyu Zhou, Miao Yu

**Affiliations:** 1 School of Materials Science and Engineering, South China University of Technology, Guangzhou 510000, China; qianlu@scut.edu.cn; 2 School of Mechanical Engineering, Sichuan University, Chengdu 610065, China; zhangkai@scu.edu.cn (K.Z.); guoxin@scu.edu.cn (X.G.); zjy822512@163.com (J.Z.)

**Keywords:** gelatin, single-molecule force spectroscopy, binding water, single-chain elasticity

## Abstract

Gelatin is an important natural biological resource with a wide range of applications in the pharmaceutical, industrial and food industries. We investigated the single-chain behaviors of gelatin by atomic force microscopy (AFM)-based single-molecule force spectroscopy (SMFS), and found that gelatin exists as long chains by fitting with the M-FJC model. By comparing the single-chain elasticity in a nonpolar organic solvent (nonane) and DI water, it was surprising to find that there was almost no difference in the single-chain elasticity of gelatin in nonane and DI water. Considering the specificity of gelatin solubility and the solvent size effect of nonane molecules, when a single gelatin chain is pulled into loose nonane, dehydration does not occur due to strong binding water interactions. Gelatin chains can only interact with water molecules at high temperatures; therefore, no further interaction of single gelatin chains with water molecules occurred at the experimental temperature. This eventually led to almost no difference in the single-chain F–E curves under the two conditions. It is expected that our study will enable the deep exploration of the interaction between water molecules and gelatin and provide a theoretical basis and experimental foundation for the design of gelatin-based materials with more functionalities.

## 1. Introduction

Green development to promote the harmonious coexistence of humans and nature is an important development trend worldwide [1,2,3]. The nonrenewable and nondegradable properties of resources such as coal, oil and natural gas have a huge impact on the environment. As the only renewable carbon source, biomass resources have attracted a large amount of attention due to their biodegradability, recyclability and environmental friendliness, and are an important way to obtain many biomass chemicals [4,5,6,7,8,9]. Gelatin is a natural water-soluble biodegradable polymer material. As a protein derived from living organisms, gelatin is widely used in the food, pharmaceutical and photographic industries due to its excellent characteristics, such as wide sources, low price, good biocompatibility, nontoxicity and multifunctional physicochemical properties [10,11,12,13,14].

Gelatin is a mixture of high-molecular-weight peptides obtained by the partial hydrolysis of collagen, a macromolecular protein with many amino acids arranged in long chains [15]. The composition of gelatin is more complex, with molecular weights ranging from a few thousand to 100,000 with a wide distribution. Previous studies have shown that gelatin is insoluble in cold water but soluble in hot water, forming a thermally reversible gel (about 30 to 40 °C) [16]. In water, the gelatin chains assume a coiled form, and cooling causes a conformational change to form a high degree of helix [17]. Both helical conformation and coiled conformation exist in gelatin products, and the ratio of the two conformations has an important influence on the mechanical properties of the final material [18]. Since gelatin molecular chains are prone to hydrogen bonding, this results in a brittle gelatin film that is prone to fracture, which in turn promotes plasticizers (ethanol, sorbitol, glycerol, etc.) being used to improve the plasticity of gelatin [19,20]. It can be seen that the study of molecular conformation and intermolecular interactions is important for the further development and utilization of gelatin systems. However, to date, the physicochemical properties of gelatin have been studied by macroscopic methods [21,22], and many molecular mechanisms are still unclear due to the complexity of gelatin composition. Therefore, it is urgent to investigate and characterize the properties of gelatin at the molecular level.

Studies at the molecular level rely on advances in science and technology, particularly advances in tips technology that have made the direct observation and manipulation of single-atom motion on surfaces a reality. Through the atom force microscopy (AFM)-based single-molecule force spectroscopy (SMFS) technique, stronger external forces are applied to the AFM tip, forcing it to interact with the polymer on the substrate and forming a polymer bridge between the substrate and the AFM tip. The intra- and intermolecular interactions of the polymer are obtained directly through the stretching of the polymer bridge by the AFM tip in different surrounding environments. A detailed review of AFM in terms of the fundamentals of force spectroscopy, sample preparation, data analysis and applications, and its future, is presented by Park and his coworkers [23]. Gaub et al. found that the elongation of the polymer chains was governed by the distortion of the bond angles at higher forces through SMFS. Polymers with specific structures undergo significant conformational changes, and such conformational changes have been found to be reversible [24,25,26,27]. In the biological field, combining molecular cell biology with SMFS, the complexity of cell or bacterial adhesion is explored to clarify the mechanism by which cell signaling processes enhance adhesion [28,29,30,31]. In the field of physical chemistry, the single-chain mechanical behavior of polymers in different environments can be obtained by SMFS to explore the interactions between single polymer chains and the surrounding environment [32,33,34,35,36,37]. These studies have provided a significant theoretical basis for exploring the patterns of polymers at the microscopic level and, therefore, can also be used for the study of degradable polymer systems.

The single-chain properties of polymers are one of the important factors that determine their macroscopic material properties. As a typical degradable bio-based polymer, gelatin has a complex molecular structure with both acidic and basic properties, and is an amphoteric substance whose single-chain properties have not been investigated. In this paper, the single-chain elasticity of gelatin can be obtained by AFM-based SMFS. By comparing the single-chain elasticity of gelatin nonpolar organic solvents and DI water, the interaction between water molecules and gelatin is explored, which is expected to provide a theoretical and experimental basis for the design of gelatin-based materials with more functionalities.

## 2. Materials and Methods

### 2.1. Materials

Gelatin (G7041, from cold water fish skin, solid) was purchased from Sigma-Aldrich (St. Louis, MO, USA). Octane and other experimental chemicals were purchased from Chengdu Kelong Chemical company (Chengdu, China). All chemicals were analytically pure and were not otherwise processed prior to use. The water used in this paper as deionized (DI) water (>18 MΩ·cm). 

### 2.2. Sample Preparation

The gelatin powder was dissolved in DI water (60 °C for 1 h) with a concentration of 5 mg L^−1^. A new quartz slide was treated with piranha solution for 30 min to remove impurities on the surface. Subsequently, the quartz substrate was rinsed with a large amount of DI water to remove excess piranha solution residue and then dried in an oven for further use. A few drops of the gelatin solution were adsorbed on the treated substrate for 30 min, followed by rinsing with abundant DI water to remove the polymer that was not firmly adsorbed. Finally, the adsorbed substrate was blown dry and used immediately for the SMFS experiment.

### 2.3. Force Measurements

The experimental instrument in this paper was an atomic force microscope manufactured by Asylum Research (MFP-3D) (Santa Barbara, CA, USA). The AFM tip was a Bruker MSCT, and the elastic coefficient of the silicon nitride tip could be measured by the thermal vibration method, which was 30–40 pN nm^−1^. AFM tips were processed by a vacuum low-temperature plasma treatment for 1 min before use to remove possible surface impurities. Previous studies have shown that the single-chain elasticity of the polymer is independent of the stretching rate for polymers that do not have a specific structure [38,39]. Therefore, the experiment rate in this paper was 2.0 μm s^−1^, and the temperature was room temperature (26 ℃). Before each experiment, the AFM tips and samples were reconstituted to ensure that the single-chain elasticity of the gelatin was obtained. In order to ensure the accuracy of the experimental data, single-chain elasticity experiments of gelatin under the same conditions were performed at least three times.

## 3. Results and Discussion

### 3.1. The Single-Chain Inherent Elasticity of Gelatin

The single-chain elasticity of polymers was determined by a combination of their molecular structure and the interaction with the surrounding environment. When there was no interaction between the polymer and the surrounding environment (or the interaction was negligible), the single-chain elasticity was the single-chain inherent elasticity. In other words, the single-chain inherent elasticity of a polymer was determined by the molecular structure of the polymer. Although a vacuum environment is ideal for obtaining the single-chain inherent elasticity of a polymer, this could not be achieved due to the limitations of our equipment. In nonpolar organic solvents, there are only weak van der Waals interactions between the polymer and solvent molecules, which can be ignored under the equipment noise level (about 10pN), making it a good alternative to vacuum for obtaining the single-chain inherent elasticity of gelatin [40]. 

**Figure 1:**
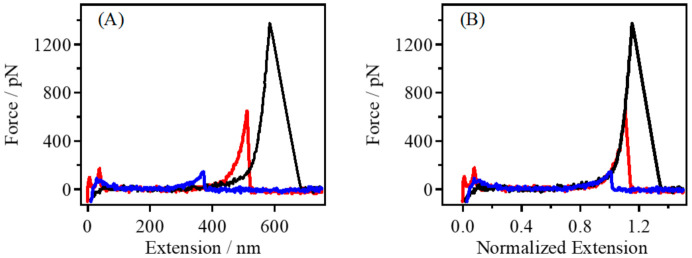
(**A**) Typical single-chain F–E curves of gelatin obtained in nonane, and (**B**) the normalized single-chain F–E curves of those shown in (**A**).

In the present work, the nonpolar organic solvent nonane was used as an alternative to the vacuum environment. As shown in Figure 1A, multiple typical force–extension (F–E) curves of gelatin in a nonane environment were obtained. It can be seen that the force value increased monotonically with the increasing stretching distance. At the top of the force value, the polymer bridge between the AFM tip and the substrate broke under external forces, and the force value quickly returned to the noise level. The F–E curves represent the process by which a polymer is captured by an AFM tip and stretched to rupture. Due to the wide molecular weight distribution of gelatin and the random stretching point of the AFM tip, the contour length of the obtained F–E curve varied. To compare the F–E curves for different contour lengths, the curves corresponding to the same force value (300 pN in this paper) were normalized, as shown in Figure 1B. It could be found that the F–E curves of gelatin obtained in nonane could be superposed well, indicating that the single-chain inherent elasticity of gelatin was obtained.

In order to further verify the correctness of the single chain elasticity of gelatin, the experimental results were compared with classical theoretical results [41,42]. According to previous research results, there are some physical models that can be successfully used to describe the stretching process of polymers. In this work, the modified freely joint-chain (M-FJC) model was used to describe the single-chain elasticity of gelatin. The M-FJC model has been successfully used to describe the single-chain elasticity of a variety of polymers in different surrounding environments [38,43,44]. The M-FJC model based on the Langevin function treats polymers as statistically independent chains of fragments. It is worth noting that the Langevin function itself (the FJC model) is based on strict statistical mechanics, but the modified model should be considered as an empirical model. The end-to-end distances can be calculated as a function of the M-FJC model:

(1) $$R\left(F\right)=\{coth[\left(Fl_{k}\right)/\left(k_{B}T\right)]-\left(k_{B}T\right)/\left(Fl_{k}\right)\}\left(L+nF/K_{segment}\right)$$

**Figure 2:**
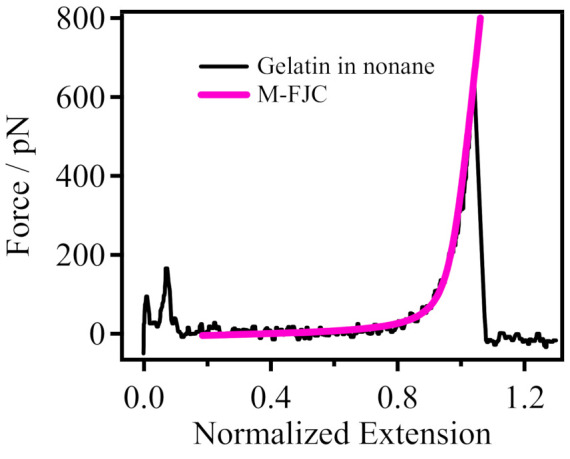
Comparison of the theoretical model (M-FJC) and experimental F–E curve of gelatin obtained in nonane.

In Equation (1), *R* represents the extension of the polymer chain (end-to-end distance), and *F* is the external force applied to the individual polymer chain. The length of the segments is the Kuhn length (*l_k_*), and the segments that can be deformed under stress are freely connected together. *k_B_* is the Boltzmann constant, *T* is the temperature, *L* is the contour length of the polymer chain, and *n* is the number of segments being stretched, which can be obtained from *L/l_k_*. The deformation of the segments is characterized by the elasticity of the segments, *K_segment_*. The parameters *l_k_*, *L* and *K_segment_* are allowed to vary freely. This model will provide an end-end distance (*R*) corresponding to a given external force (*F*) and a set of parameters (*l_k_*, *L* and *K_segment_*) [39]. As shown in Figure 2, the theoretical model (pink line) can describe the experimental curve well at *L* = 1 nm, *l_k_* = 0.67 nm and *K_segment_* = 14,000 pN/nm, which indicates that the obtained F–E curve is the inherent elasticity of single gelatin chain in a nonpolar organic solvent. Furthermore, since gelatin has no fixed structure, it is intuitively assumed that gelatin may be amorphous, but our experimental results demonstrate that gelatin exists as a single long chain.

### 3.2. The Single-Chain Elasticity of Gelatin in DI Water

**Figure 3:**
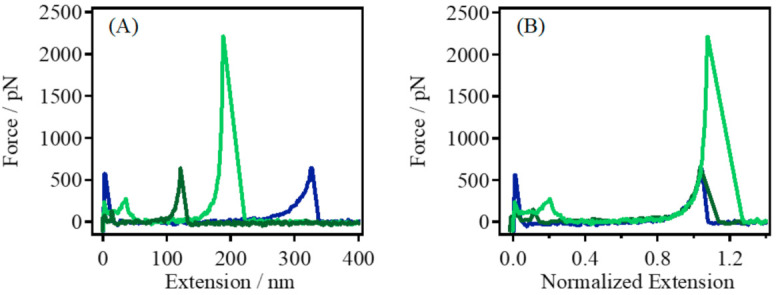
(**A**) Typical single-chain F–E curves of gelatin obtained in DI water, and (**B**) the normalized single-chain F–E curves of those shown in (**A**).

Gelatin is an amphiphilic polymer with good water solubility, and most of its application scenarios are in water; therefore, the single-chain elasticity of gelatin in DI water was studied. As shown in Figure 3A, several F–E curves of gelatin in DI water were obtained, and the F–E curves could be effectively superposed after normalization (Figure 3B), indicating that the single-chain elasticity of gelatin in DI water was obtained.

**Figure 4:**
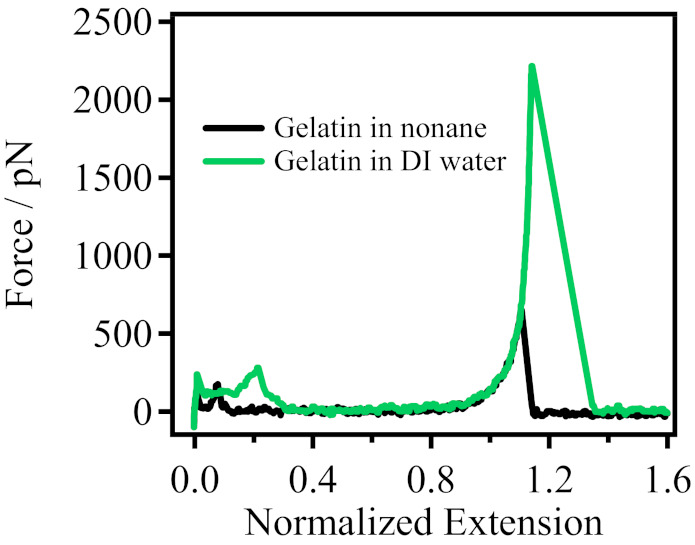
Comparison of gelatin in nonane (black line) and DI water (green line).

To the best of our knowledge, in general, polymer chains are pulled from the substrate into nonpolar organic solvents through an interface, where they undergo a “dehydration” reaction. Thus, polymers can exhibit their inherent elasticity in nonpolar organic solvents. However, it is interesting to note that our results showed almost no difference in the single-chain elasticity of gelatin in DI water and in nonane, as shown in Figure 4. The dissolution of gelatin is special in nature, and the dissolution process is slow, requiring two processes. First, gelatin powder was immersed in DI water for a period of time for limited dissolution, then put into a 60 °C water bath for 1 h for infinite swelling and dissolution, and finally was made into a single molecule sample. Therefore, the single-chain elasticity of gelatin obtained in DI water due to sufficient dissolution contained the single-chain elasticity of gelatin itself and the interaction between single gelatin chain and water molecules. Previous studies have shown that the single-chain elasticity of polymers in an undisturbed environment is their inherent elasticity [40]. However, for single gelatin chains dissolved in warm DI water, the interactions of single gelation chain and water molecules are strong, and the binding water may become part of the body of the single gelation chain. Furthermore, the large size of the nonane molecule (nine carbon atoms) corresponds to the formation of some cavities in the interface, and the overall structure of the solvent is loose, with large voids between solvent molecules [45]. The binding water molecules of the single gelatin chain can pass through the cavity directly into the nonpolar solvent (nonane) without being removed under interfacial interaction. Thus, the “single-chain inherent elasticity” obtained in nonane is the combination interactions of the single gelatin chain itself and its binding water molecules. In addition, the experimental temperature was 26 °C in this paper, which is not enough for single gelatin chains to continue to form binding water bridges with water molecules. Therefore, the single chain elasticity exhibited by gelatin in DI water was almost indistinguishable from that in nonane.

The single-chain inherent elasticity of gelation and the interactions between a single gelation chain and water molecules were studied by SMFS. When the single-chain enthalpic elasticity was the same, the different interactions between single polymer chains and the different surrounding environments could be obtained by calculating the area difference of the F–E curves. When the entropic elasticity was the same, the rigidity of polymer single chains could be obtained by M-FJC fitting, and the differences could be compared to determine the differences in single chain behavior caused by the molecular structure. When the entropic elasticity was the same, the rigidity of single polymer chains could be obtained by M-FJC fitting to determine the difference in the single chain behavior caused by molecular structure. Therefore, a reasonable experimental design would probe deeply into the interaction and mechanism of the interaction of the polymer itself, as well as with the surrounding environment. This method can be further extended to study the mechanism of the interaction between other natural and synthetic polymers and the environment.

## 4. Conclusions

In this paper, the single-chain elasticity of gelatin was obtained for the first time. The fitting of the M-FJC model showed that the theoretical curve could describe the experimental results well, proving that gelatin exists in the form of long chains. Interestingly, the single chain elasticity of gelatin obtained in a nonpolar organic solvent (nonane) was almost no different from that in DI water. There are two main reasons for this result: (1) Gelatin has a requirement for the temperature of the water in which it is dissolved. In the experimental preparation stage, gelatin was dissolved in warm water at 60 °C, thus forming a strong interaction between single polymer chains and water molecules. Due to the solvent size effect, larger nonane molecules formed some cavities at the interface, and the overall solvent was loosened, allowing the gelatin to pass through the solvent molecules without being dehydrated. (2) The experimental temperature as 26 °C, which is not enough for gelatin to continue interacting with water molecules to form binding water. Our experimental results are of great value for an in-depth study of the interaction between gelatin and water molecules. By exploring the interaction and mechanism between gelatin and water molecules (or other environmental molecules), we provide insight into the changes in gelatin properties and environmental responsiveness, and a theoretical basis for the design and application of gelatin-based composites.

## Data Availability

Data generated or analyzed during this study are included in this published article.
